# A dynamic systems view of habits

**DOI:** 10.3389/fnhum.2014.00682

**Published:** 2014-09-02

**Authors:** Nathaniel F. Barrett

**Affiliations:** Mind-Brain Group, Institute for Culture and Society, University of NavarraPamplona, Spain

**Keywords:** dynamic systems, habits, multistability, hysterisis, learning

This paper explores some of the insights offered by a dynamic systems approach into the nature of habits. “Dynamic systems approach” is used here as an umbrella term for studies of cognition, behavior, or development as systems of elements that change over time (e.g., Thelen and Smith, 1994, 2006), while “dynamical systems” is reserved for studies that use differential equations to describe time-based systems (e.g., Schöner and Kelso, 1988; Tschacher and Dauwalder, 2003). The following discussion draws primarily from the coordination dynamics research of Kelso (1995, 2012), which stems from Haken's theory of synergetics (1977, 2003). However, the view of habits presented here is more of an interpretive application than a literature review, as the work on which it draws does not address habits explicitly. Perhaps this is because conventional notions of habit are too broad and loose to be captured succinctly in dynamic terms. Dynamical studies of human behavior have focused on more specific capacities such as motor coordination (Thelen et al., 1987), perception (Tuller et al., 1994), and learning (Kostrubiec et al., 2012). Yet this variety of applications suggests that the scope of the dynamic approach overlaps significantly with the domain of habits, so that dynamic concepts could be used to challenge and refine our conventional notions of habitual behavior. Accordingly, the goal of this paper is to raise questions about the nature of habits rather than present a comprehensive scientific theory.

For a dynamic systems approach, stability is “the central concept” (Schöner and Kelso, 1988, p. 1515). The “essential issues are the stability of the system, as indexed by the behavior of some collective measure of the multiple components, and the changes in stability over time” (Thelen and Smith, 2006, p. 289). Intuitively, it would seem that the characteristic stability or stabilities of a system—its preferred states—are its habits. But the connection between stable states and habitual behavior is not as straightforward as it appears. The preferred states of a dynamic system are not simply “built in”; rather they depend on the interactive dynamics of the system's components as well as the interactive couplings of the system with its environment. The following discussion explores the implications of four features of dynamic system stability for our understanding of habit: (1) stability is relative to timescale, and system stabilities at different timescales are interdependent; (2) the attractor landscape describing the characteristic stabilities of a system can be altered by various control parameters, including situational parameters; (3) systems can have multiple stabilities, such that the stability they exhibit at any given time may depend on their recent history; (4) learning processes tend to affect a whole cluster of interrelated stabilities and not just one stability in isolation.

In light of these features of dynamic stability, it seems that there is no straightforward way to map conventional notions of personal habits onto stabilities of the human person considered as a nested dynamic system of body, brain, and environment (Chiel and Beer, 1997). Should we consider as habits only the “intrinsic” stabilities of brain and body, regardless of the variety of behaviors that can arise from these stabilities in different situations? Or should we only consider as habits those patterns of behavior that are regularly observed within a certain type of situation, regardless of how differently these patterns might be assembled at the body-brain level? From the dynamic perspective, stability and change are ubiquitous features at every level or spatiotemporal scale of description. Thus, it seems arbitrary to apply the term “habit” only to one kind or level of stability, and perhaps this explains why the term is seldom used in dynamic systems literature. Yet it could be argued that this multilevel complexity is an advantage, as it can be used to challenge conventional notions of habit in interesting ways. Let us suppose that for any level of human behavior that can be described as a dynamic system, the stabilities or preferred states of that system—its “attractor landscape”—are at least analogous to habits, and should be considered as such. What is revealed by this broader, dynamic perspective?

First, this view calls into question the usual timescale of habits, which is typically restricted to stable features of personality and behavior on the timescale of months or years (Lewis, 2000). From a dynamic perspective, these stabilities are in principle no different from stabilities at faster and slower timescales. Moreover, different timescales of stability are interrelated: while the habits of any given timescale are shaped by the “deeper” habits of a slower timescale, they also can lead to changes at this deeper level. In other words, the “force of habit” is not one-way: habits are shaped by the behaviors that they themselves constrain. For example, mood is shaped by habits of personality, while a string of similar moods can lead to changes of personality. And though it may seem strange to think of moods as temporary emotional habits on the timescale of hours or days, their way of shaping and being shaped by emotional states on the timescale of seconds or minutes is analogous to the relationship between personality and mood. Likewise one can treat emotional states as habits that contribute to the attractor landscapes for thoughts and sensorimotor activity at an even faster timescale, while personality itself can be seen as evolving over the very “deep” landscape of developmental habits (Thelen and Smith, 2006). Thus, a dynamic view opens up a wider range of timescales across which the concept of habit might apply. But more importantly, even if we choose to restrict habit to just one of these levels, the interaction of timescales suggests that our understanding of any one level should draw upon at least two neighboring levels (Kelso, 1995), if not the entire nested system.

Second, a dynamic view of habit will include regular variations of the attractor landscape that occur in relation to changing parameters. In some cases these variations can be represented by a bifurcation diagram that shows how the attractor landscape changes in relation to a single control parameter (Kelso and Engstrom, 2006, pp. 124–137). Most human behaviors, however, require that multiple parameters are taken into account. Now, provided that the important variables and parameters for describing characteristic variations of a certain behavior for an individual can be determined (a very difficult task, in most cases), one could, in theory, construct a comprehensive “habit topology” for that behavior: a map of how that behavior's preferred states change in relation to various parameters. Notice that, depending on the parameters involved, some stabilities of this habit topology will be visited by the system more or less regularly. For instance, in the case of quadruped motion, assuming that the parameter of speed varies regularly over its natural range, a quadruped (e.g., horse) regularly visits the various gaits (walk, trot, gallop) that make up the preferred states of its habit topology (Hoyt and Taylor, 1981; Schöner and Kelso, 1988, p. 1516). However, for some behaviors, especially those that are sensitive to multiple parameters, certain regions of the habit topology can remain “hidden” because the required values of the relevant control parameters are rarely if ever encountered. Imagine, for example, that a person who never dances might be found, on one occasion, happily dancing the night away. Conventionally speaking, this behavior is not habitual. But from a dynamic view, it is hard to say. Perhaps on that occasion the person encountered just the right combination of circumstances—excellent mood, pleasurable company, Afro-Cuban music, fantastic mojitos, etc.—that made, for that person, dancing a very deep (and enjoyable) stability. If these circumstances will regularly facilitate the same behavior for that person, cannot we say that dancing is habitual for them in those circumstances? The point here is not to insist on this characterization, but to question our conventional understanding of habits, which typically involves assumptions about “normal” circumstances. Are habitual behaviors only rightfully considered as such if we regularly encounter the circumstances that facilitate their expression? If not, how would we determine the unexpressed or latent habits of a person, independently of circumstances?

Third, it is important to consider that the attractor landscape of a particular behavior commonly has multiple stabilities even within restricted parameter values (Schöner and Kelso, 1988, p. 1518). This phenomenon of multistability implies that “habitual behavior” may not be always the same even when all relevant variables are the same. In such cases, which stability describes the habit? The stability into which the system enters may depend on its recent history or on a symmetry breaking. For example, as represented by the much-studied HKB model (Chemero, 2009), coordinated finger movement—waving the two index fingers in time with a metronome, where tempo or speed is the main control parameter—exhibits bistability at slower speeds and monostability at faster speeds (Kelso, 1995). Within the slower, bistable regime, which of the two stable states the system exhibits may depend on its historical trajectory: for instance, if the system has just entered the bistable regime from the monostable regime, it will remain in the preferred state of the latter. Although coordinated finger movement may not seem representative of human behavior, coordination dynamics and its telltale characteristics—such as multistability—have been found in a wide range of behavioral and neural systems (Schöner and Kelso, 1988; Kelso, 2012), suggesting an important implication: perhaps what a person presently exhibits as habitual behavior within certain circumstances does not uniquely characterize what is habitual for that person *even within those circumstances*, but must also be understood as a result of a particular historical trajectory. The importance of history is also indicated by the phenomenon of hysteresis (Haken, 2003, pp. 8–9), i.e., when the state or behavior of a system is influenced by the residual stability of an antecedent regime. Thus, certain behaviors might persist even after the attractor landscape has changed so that they are no longer “habitual” within the current context.

Finally, if we define learning in terms of alterations to the attractor landscape for a particular behavior such that some states are newly stabilized while others are destabilized (Kostrubiec et al., 2012), the interrelatedness of habits as implied by the previous three points indicates that habits are rarely altered in a piecemeal fashion: learning affects entire clusters of habits that are composed of shared components. That is, learning affects an entire “habit space,” and not just individual habits in isolation (Kelso, 1995, pp. 159–186). This further complicates the picture of personal habits, as well as the kinds of learning strategies we should adopt to change them. For instance, it suggests that certain habitual behaviors that are difficult to target can be altered indirectly by focusing on other, related behaviors. It also suggests that in many cases—especially where a complex task demands a finely articulated set of behaviors—training should focus on shaping the overall “habit space” rather than each individual behavior. Moreover, as implied by the definition of learning just given, the dynamical approach suggests that habits are a necessary condition for learning and not just a product—contrary to the so-called “blank slate” model (Kostrubiec et al., 2012).

In conclusion, we see that a fairly simple definition of habit in terms of dynamic stability yields a number of insights that question our conventional notion of personal habits as slow-changing, context-independent, uniquely repetitive, and discrete behaviors. At the same time, a dynamical view can give an account of the relative fixity of behaviors that we take to be habitual in the conventional sense. However, by showing that this fixity is dynamically and relationally constituted, a dynamical view reveals the elaborate context in which all habits are embedded. Moreover, human habits appear to be instances of a very general pattern: all living systems exhibit self-organized stabilities that reduce their degrees of freedom, producing robust but flexible repertoires of behavior.

## Conflict of interest statement

The author declares that the research was conducted in the absence of any commercial or financial relationships that could be construed as a potential conflict of interest.
